# Underwater Vehicle Positioning by Correntropy-Based Fuzzy Multi-Sensor Fusion

**DOI:** 10.3390/s21186165

**Published:** 2021-09-14

**Authors:** Nabil Shaukat, Muhammad Moinuddin, Pablo Otero

**Affiliations:** 1 Institute of Oceanic Engineering Research, University of Malaga, 29010 Malaga, Spain; pablo.otero@uma.es; 2 Department of Electrical and Computer Engineering, King Abdulaziz University, Jeddah 21589, Saudi Arabia; mmsansari@kau.edu.sa; 3 Center of Excellence in Intelligent Engineering Systems, King Abdulaziz University, Jeddah 21589, Saudi Arabia

**Keywords:** underwater vehicle, fuzzy, multi-sensor fusion, correntropy, positioning, Kalman filtering, underwater robotics

## Abstract

The ability of the underwater vehicle to determine its precise position is vital to completing a mission successfully. Multi-sensor fusion methods for underwater vehicle positioning are commonly based on Kalman filtering, which requires the knowledge of process and measurement noise covariance. As the underwater conditions are continuously changing, incorrect process and measurement noise covariance affect the accuracy of position estimation and sometimes cause divergence. Furthermore, the underwater multi-path effect and nonlinearity cause outliers that have a significant impact on positional accuracy. These non-Gaussian outliers are difficult to handle with conventional Kalman-based methods and their fuzzy variants. To address these issues, this paper presents a new and improved adaptive multi-sensor fusion method by using information-theoretic, learning-based fuzzy rules for Kalman filter covariance adaptation in the presence of outliers. Two novel metrics are proposed by utilizing correntropy Gaussian and Versoria kernels for matching theoretical and actual covariance. Using correntropy-based metrics and fuzzy logic together makes the algorithm robust against outliers in nonlinear dynamic underwater conditions. The performance of the proposed sensor fusion technique is compared and evaluated using Monte-Carlo simulations, and substantial improvements in underwater position estimation are obtained.

## 1. Introduction

Precise seabed mapping is the ultimate requirement for extracting minerals and other natural resources from the ocean. Underwater vehicles play important roles in mapping and exploration, but their precision is highly affected by the noise conditions in the ocean environment. Moreover, the main navigation sensors of the underwater vehicle, such as gyros and accelerometers, suffer from drift and bias. As the worldwide satellite-based positioning system that uses radio frequency cannot be accessed underwater, an alternate means of communication based on acoustic positioning systems is usually employed, making precise location determination of vehicles considerably more difficult than it is for land vehicles. Ray bending, reflection, and the multi-path effect are all serious barriers in determining a vehicle’s underwater position [1].

On the other hand, multi-sensor fusion algorithms based on Kalman filtering require complete knowledge of system model and noise characteristics, which is difficult to obtain in underwater environments. Typically, for the system process model, the deterministic component of the underwater vehicle is often derived using kinematic principles, whereas the stochastic element of the model is represented by noises, which are mostly influenced by modeling errors and nonlinearity. Likewise, the stochastic portion of the measurement model is heavily impacted by sensor characteristics and reliability. In a practical underwater vehicle navigation scenario, the system process and measurement noise covariance are unknown, and incorrect values cause compromised position estimation and divergence. The best estimation of underwater vehicle position is only possible with prior knowledge of noise covariance [2]. Thus, correct adaptation to the underwater noise environment is a key requirement for getting a precise position to mapping the seafloor.

Fuzzy logic has provided a simple solution for adaptation of noise covariance using expert knowledge [3,4,5]. It has the ability to define complex nonlinear equations with a simple linguistic rule base. Most previous research studies [6,7,8] have been based on matching theoretical and actual covariance based on the difference, but this method does not give accurate results in the heavy outliers in the stochastic part of the model.

Information-theoretic learning (ITL) has been successfully utilized to test nonlinear similarity based on correntropy, particularly for noisy outlier environments [9]. As a nonlinear similarity measure, correntropy shows the closeness of two random variables with the given kernel size. Furthermore, it can preserve nonlinear features, as well as high-order moments [10]. However, the existing correntropy-based works are lacking the advantages of using fuzzy logic. These advantages of fuzzy logic and correntropy motivated us to propose new algorithms and drived us to answer a major research question: Can we use correntropy’s strengths to improve underwater vehicle navigation performance in the presence of nonlinearity and outliers?

### 1.1. Review of Previous Work

Different types of adaptive Kalman filtering for underwater navigation applications have been developed and used since the advent of the Kalman filter [11,12]. Initially, Mehra laid the foundation by proposing four state-of-the-art approaches intended to address a situation in which the system and measurement noise covariance matrices cannot be known during the design phase, or to correct for scenarios in which both covariance matrices change over time [2]. These four techniques are Bayesian, correlation, Maximum Likelihood Estimation (MLE), and covariance matching. These techniques have been applied in various land, air, and space applications [13,14,15,16,17].

Many research investigations are being done on underwater vehicle positioning, and navigation evolves during off-shore resources exploration. A study proposed modifications of multi-model Kalman filters for underwater navigation by using the probabilistic data association theory and claimed to improve the navigation accuracy [18]. Overall, the method is computation- and memory-intensive due to the use of multiple Kalman filters, and requires previous steps of states for autocorrelation calculation. Through probability calculations, they dynamically determine the most efficient navigation routes. Another piece of research conducted recently used the MLE and RTS smoother for process and measurement noise adaptation to eliminate range error [19]. According to them, using only one acoustic beacon in this solution makes it more cost-effective. However, the RTS smoother can significantly increase the computational time and memory requirement. In addition, the main problem of the MLE method is high sensitivity to outlier auxiliary data.

The improved Sage–Husa adaptive Kalman filter was claimed to enhance the underwater navigation accuracy of a tightly coupled, strapped down inertial navigation system (SINS) and Doppler velocity log (DVL)-based system [20]. The method employed the forgetting factor for memory optimization and variable sliding window for decreasing computational time. Another study proposed a two-stage adaptive information filter that used an ultra shot baseline (USBL) with DVL for estimation of an unknown sea current [21]. Their design is based on two information filters—one is a standard information filter for estimation of states, and the other is based on the sequential least squares algorithm for estimating the velocity of unknown sea currents. Furthermore, they introduced the forgetting factor for fast processing of new data. However, running two-stage filters can add computational load, which is not discussed in the study.

Recently, improvement in the variational Bayesian approximation-based adaptive Kalman filter (VB-AKF) was proposed by using asynchronous auxiliary sensor measurements [22]. They claimed that this technique significantly reduces RMSE for position estimation. In recent years, there has been growing interest in neural network-based underwater navigation. Various approaches have been proposed, and more recently, a research study used a deep recurrent neural network involving sequential learning with Long Short-Term Memory (LSTM) [23]. They claimed that their method outperformed Kalman-based solutions in terms of accuracy. However, they did not mention the training time requirement, processing load, and the total number of neurons used in the network. Another current study proposed an end-to-end navigation solution based on deep hybrid recurrent neural networks and used raw sensors data directly to estimate the location underwater vehicle [24]. An investigation that was conducted recently took advantage of Reinforcement Learning (RL) and incorporated the deep deterministic policy gradient for tuning the process noise covariance matrix online from low-cost navigational sensors [25]. Their method used the positioning error as a reward function for training RL. However, the performance of RL neural network-based algorithms are directly proportional to the training period and previous data storage in the memory.

Several authors have also suggested the use of terrain-based underwater navigation, which aims to solve the long-distance underwater navigation problem [26,27]. These terrain-based navigation solutions utilize bathymetric data, underwater topographical features, and underwater earth gravitational and magnetic profiles [28]. The concept behind terrain navigation is to construct a terrain profile map from sensor measurements, then compare it to a previous map database to get the best location estimate. For instance, a detailed study targeting underwater vehicles used multi-model adaptive estimation (MMAE) for terrain-based navigation. They utilized Principal Component Analysis (PCA) with MMAE for underwater terrain matching [29]. In recent research, neural networks were combined with terrain-based navigation [30]. They used the Rao-Blackwellized particle filter and offline trained neural network with terrain maps for pattern recognition from time-series data. However, terrain-based techniques are only useful when previous map databases are available and sensors are accurate. Moreover, carrying these map databases on underwater vehicles increase memory requirements, processing power, and computational load.

The application of the fuzzy set theory enables human experience, understanding, and rationale to be used by computer programs. Fuzzy systems have been documented in numerous control theories and state estimation filters since the invention of fuzzy logic and the accompanying mathematics, notably the fuzzy Kalman Filter. Because they do not require any mathematical model of the system, adaptive fuzzy filters are particularly effective in dealing with nonlinearity and modeling inaccuracies. Sasiadek et al. made a seminal contribution by employing fuzzy logic with a Kalman filter for adaptation of noise covariance. The research work utilized nine rules, with covariance residuals and the mean of residuals as input, for designing the exponential weighting factor [31]. A large number of existing research studies in the broader literature has used fuzzy logic for designing addition or multiplication factors to the noise covariances [5,32,33]. However, there are wider choices of fuzzy inputs available in the literature depending on the application. The popular choices of input are actual covariance, Degree of Divergence (DOD), Degree of Mismatch (DOM), the difference between the theoretical and actual covariance, and mean value of the residual [34,35]. More recently, adaptive fuzzy Kalman was proposed for spacecraft navigation based on DOM and DOD. They claimed that fuzzy-based methods require fewer computing resources than the MLE-based method [36]. Despite this, the performance of this method has not been tested with data affected by outliers, which are common in underwater environments [37,38].

### 1.2. Novelty and Contributions of the Proposed Research

The ultimate goal of this research is to take the benefits of correntropy’s strengths and to address the current need for improving underwater vehicle navigation performance in the presence of nonlinearity and non-Gaussian outliers. There is no previous underwater multi-sensor-fusion method, to the best knowledge of the authors, that augments the benefits of correntropy, fuzzy, and  Kalman filtering applied to the improvement of autonomous underwater vehicle navigation. It is aimed at improving the performance by using fuzzy logic, which has the benefit of handling nonlinearity based on expert knowledge, correntropy for robust handling of a non-Gaussian outlier, and Kalman filter for real-time minimum error variance processing. We named this algorithm FC-MSF, where FC stands for Fuzzy Correntropy and MSF for Multi-Sensor data Fusion. Another noteworthy contribution of this work is the introduction of new metrics based on correntropy, which use high-order moments for improving covariance matching adaptation. We proposed these metrics as a Degree of Similarity and Degree of Convergence that compare theoretical and actual covariance statistically, which was not the case in previous DOM and DOD. Moreover, the fuzzy logic and correntropy-based similarity measures together provide more robustness to the large outliers which are commonly present in underwater acoustic position measurements and velocity measurements by DVL. Correntropy provides a similarity measure based on kernels, specifically the Gaussian and Versoria kernels, because of their distinct advantages to the heavier tail underwater vehicle stochastic data. A simulation study has demonstrated the superior performance of the proposed FC-MSF algorithm and validated that the novel correntropy-based metrics improved outlier influenced underwater navigation in the absence of global satellite-based positioning systems.

The remaining structure of this paper is organized as follows. Section 2 provides a brief overview of the mathematical modeling of underwater vehicle sensors. This includes a discussion on both on-board and off-board sensors of the underwater vehicle. In addition to that, it briefly discusses navigation equations derived from the gyro and accelerometer. The mathematical formulation of Kalman filtering with an error dynamic model is briefly discussed in Section 3. This formulation acts as the foundation of the proposed multi-sensor fusion. Moreover, it highlights the major shortcomings of Kalman filtering. Furthermore, it highlights the major shortcomings of Kalman filtering. In Section 4, a fuzzy correntropy-based multi-sensor fusion algorithm is proposed for adaptation of unknown noise covariances. Section 5 discusses and compares the test results of the proposed correntropy-based fuzzy multi-sensor fusion with previous methods. Importantly, the suggested technique is evaluated by injecting the outlier as a shot non-Gaussian noise, which is a prominent method used by many researchers to assess the robustness of the filter [10,39,40]. Subsequently, a comparative analysis is performed, which enables the authors to assess the accuracy of the suggested method. Monte Carlo simulations indicate that the approach is technically feasible and has the potential of yielding positive results in a harsh underwater environment. In the final section, conclusions are provided.

## 2. Mathematical Modeling of Underwater Vehicle Navigation

This section is divided into two parts. The first subsection discusses mathematical models of underwater vehicle sensors, while the second subsection provides a brief overview of navigation equations.

### 2.1. Mathematical Models of Navigation Sensors

This section briefly highlights the sensors and motion model of an underwater vehicle. For details, readers may refer to [41,42].

**Figure 1:**
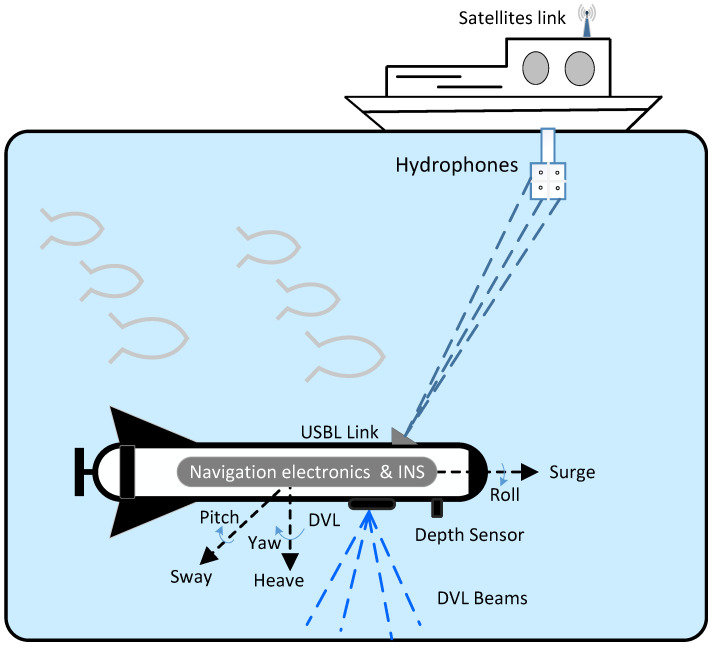
System level diagram of sensors used by vehicle while maneuvering underwater.

The system-level diagram of the underwater vehicle is shown in Figure 1.

The navigation electronics shown in Figure 1 carry the main embedded microprocessor and primary sensors used for navigation. The inertial navigation system (INS) is comprised of an inertial measurement unit (IMU) which gives data at a high rate to the processor. The IMU contains gyros and accelerometers, which are referred to as primary sensors. The low-rate secondary sensors include a depth sensor, an electronic compass, and acoustic position transceivers, which enable USBL connection with the vessel and DVL.

IMU is the most critical onboard instrument for autonomous underwater navigation. For three-axis motion, the IMU consists of three gyroscopes and accelerometers.

The three orthogonal accelerometers ${\hat{f}}_{ib}^{b}$ and gyros actual output vector ${\hat{\omega }}_{ib}^{b}$ in the body frame are modeled as

(1) $${\hat{f}}_{ib}^{b}=f_{ib}^{b}+b_{acc}+\varrho_{acc}$$

(2) $${\hat{\omega }}_{ib}^{b}=\omega_{ib}^{b}+b_{g}+\varrho_{g},$$

where the actual output of accelerometers ${\hat{f}}_{ib}^{b}$ is a combination of white noise $\varrho_{acc}$ and accelerometer bias $b_{acc}$. The actual output of gyros ${\hat{\omega }}_{ib}^{b}$ is sum of white noise $\varrho_{g}$ and gyro bias $b_{g}$. The accelerometer and gyro biases are modeled as the first-order Markov process. The subscript acc is used for noise, and the bias values belong to the accelerometer output, while the subscript *g* is used for noise and the values are used for gyro output. The unit of gyroscope output is radians per second, and that of the accelerometer is meters per second. The frames of references are represented by the superscript and subscript (i) for the inertial frame, (n) for the north-east down frame (NED), (e) is the earth-centered, earth-fixed frame (ECEF) frame, and (b) is the body frame. The relationship between the frames is represented by subscripts and superscripts. For instance, $\omega_{ib}^{b}$ shows an angular velocity of frame (b) with respect to (i) represented in the (b) frame [41].

The acoustic positioning system is the second most important instrument on the underwater vehicle. Acoustic transponder beacons are used by underwater vehicle navigation systems to determine the vehicle’s position. An underwater vehicle uses the Ultra-Short Baseline (USBL) system to position itself relative to a surface vessel that has GPS-calibrated transponders.

The actual output of the USBL acoustic positioning system ${\hat{p}}_{h}$ is represented as

(3) $${\hat{p}}_{h}=p_{h}+b_{h}+\varrho_{h},$$

where ${\hat{p}}_{h}$ is a combination of the true acoustic position $p_{h}$, time-varying bias $b_{h}$, and white noise $\varrho_{h}$.

The DVL measures the speed of the vehicle with respect to the bottom by measuring the change in acoustic frequency [43]. The output of DVL ${\hat{v}}_{dvl}$ measurement can be modeled as a sum of the actual velocity measurement vector $v_{dvl}$, noise $\varrho_{dvl}$, and bias $\varrho_{dvl}$, given as

(4) $${\hat{v}}_{dvl}=v_{dvl}+b_{dvl}+\varrho_{dvl}.$$

The depth of the underwater vehicle is measured by a pressure sensor which has an almost linear relationship. The actual depth sensor output ${\hat{h}}_{d}$ is a combination of true output ${\hat{h}}_{d}$ with added noise $\varrho_{d}$, represented as

(5) $${\hat{h}}_{d}=h_{d}+\varrho_{d}.$$

The vehicle’s attitude is determined by an electronics compass, which senses the magnitude and direction of the Earth’s magnetic field [44]. The actual output electronic compass $q_{m}$ is a product of true output $q_{m}$ and noise. It is represented as

(6) $${\hat{q}}_{m}=q_{m}\varrho_{m}.$$

### 2.2. Navigation Equations

A strapdown INS configuration due to its weightlessness is widely used in underwater vehicles. In this configuration, navigation equations are calculated in an embedded microprocessor, that uses IMU data to determine the vehicle’s attitude, velocity, and position [45].

The rate of change of attitude of the vehicle is obtained from the gyro angular velocity represented by a quaternion ${\dot{q}}_{b}^{e}$ by the following equation:

(7) $${\dot{q}}_{b}^{e}=\frac{1}{2}\Omega_{g}^{b}q_{b}^{e},$$

where $q_{b}^{e}$ is the attitude for which an initial value is required for the first-time calculation, and it is comprised of four elements: $\left[q_{1}\;q_{2}\;q_{3}\;q_{4}\right]$. The scalar component is $q_{1}$, while the vector part is $q_{i}$, *i* = 2, 3, 4. The $\Omega_{g}^{b}$ is a skew symmetric matrix. The attitude is obtained by numerical integration of ${\dot{q}}_{b}^{e}$.

The differential equation for underwater vehicle velocity $\dot{v^{e}}$ is determined from an accelerometer measurement $f^{b}$, angular velocity $\omega_{ie}^{e}$, and the gravity vector of earth $g^{e}$ is expressed as [46,47]

(8) $$\dot{v^{e}}=R_{b}^{e}f^{b}-2\Omega_{ie}^{e}v^{e}+g^{e},$$

where $R_{b}^{e}$ is the rotation matrix. $\Omega_{ie}^{e}$ is a skew symmetric matrix. Numerical integration is required for obtaining the current velocity of the vehicle.

The velocity $v^{e}$ obtained in the previous equation is related to the position ${\dot{p}}^{e}$ by the following differential equation:

(9) $${\dot{p}}^{e}=v^{e}.$$

For more details of kinematic equations, readers may refer to [42,45,48].

## 3. Shortcomings of Kalman Filtering with Error Dynamic Model

This Section begins by giving a brief overview of Kalman filtering with error dynamics, and the last part discusses Kalman filtering’s shortcomings. The state space representation is based on the error dynamic model $\dot{x}$, which is the difference between the true state $\dot{\tilde{x}}$ and estimated state $\dot{\hat{x}}$, written as

(10) $$\dot{x}=\dot{\tilde{x}}-\dot{\hat{x}},$$

where the true states are obtained from INS primary sensors. The complete error state vector of position p, velocity v, attitude q, gyro bias $b_{g}$, accelerometer bias $b_{acc}$, and acoustic fix bias $b_{h}$ is given as

(11) $$x={\left[\begin{array}{cccccc} p & v & q & b_{g} & b_{acc} & b_{h} \end{array}\right]}^{T}.$$

The nonlinear dynamics of the underwater vehicle state $\dot{x}\left(t\right)$ and measurement output equation z(t) are represented by

(12) $$\dot{x}\left(t\right)=f\left(x\left(t\right),u\left(t\right),t\right)+w\left(t\right)$$

(13) z(t)=h(x(t),t)+v(t),

where f and h are nonlinear functions. In the case of EKF, they are transformed linearly by using Taylor approximation [F] and [H]. The states and measurements are corrupted by process noise w(t) and measurement noise v(t), respectively.

EKF begins by the prediction or time update step that includes the error state $x_{k+1}^{-}$ and error state covariance $P_{k+1}^{-}$ prediction [47]. The superscript minus ${}^{-}$ and superscript plus ${}^{+}$ denote the a priori and posteriori states.

(14) $$x_{k+1}^{-}=\Phi_{k}x_{k}^{-},$$

where $\Phi_{k}$ is the state transition matrix which depends on error states in discrete form.

(15) $$P_{k+1}^{-}=\Phi_{k}P_{k}^{+}\Phi_{k}^{⊤}+Q_{k},$$

where $Q_{k}$ is the covariance of process noise $v_{k}$.

In the next step, the filter performs corrections or a measurement update, in which the posteriori error state $x_{k}^{+}$ and error covariance $P_{k}^{+}$ are computed using Kalman gain $K_{k}$; given by following equations:

(16) $$K_{k}=P_{k}^{-}H_{k}^{⊤}{\left(H_{k}P_{k}^{-}H_{k}^{⊤}+R_{k}\right)}^{-1}$$

(17) $$x_{k}^{+}=x_{k}^{-}+K_{k}\left(z_{k}-H_{k}x_{k}^{-}\right)$$

(18) $$P_{k}^{+}=\;\left(I-K_{k}H_{k}\right)P_{k}^{-},$$

where $R_{k}$ is the covariance of measurement noise $w_{k}$.

The complete corrected navigation state ${\hat{x}}_{k}^{+}$ can be written as the sum of the error estimate from Equation (17) and prior full state estimate ${\hat{x}}_{k}^{-}$ as

(19) $${\hat{x}}_{k}^{+}={\hat{x}}_{k}^{-}+x_{k}^{+}.$$

Nevertheless, when the measurements are contaminated by non-Gaussian noise, such as outliers or impulsive noise inference, EKF will perform poorly and even diverge [49]. The term $\left(z_{k}-H_{k}x_{k}^{-}\right)$ is known as innovation. It is the difference between the measurement error vector $z_{k}$ and its predicted error vector $H_{k}x_{k}^{-}$. If the heavy outliers impact measurement or process modeling by including errors caused by nonlinearity, the innovation term will produce erroneous results, causing the filter to diverge. Specifically, in underwater conditions, a major limitation for using Kalman filtering is the limited prior knowledge of process noise and measurement noise covariances. Incorrect initialization of covariance can cause filters to diverge. Moreover, the statistics of noise can change according to underwater conditions. Consequently, the adaptation of process and measurement covariance is necessary to get explicit navigation accuracy of the underwater vehicles.

## 4. Correntropy-Based Fuzzy Multi-Sensor Fusion

**Figure 2:**
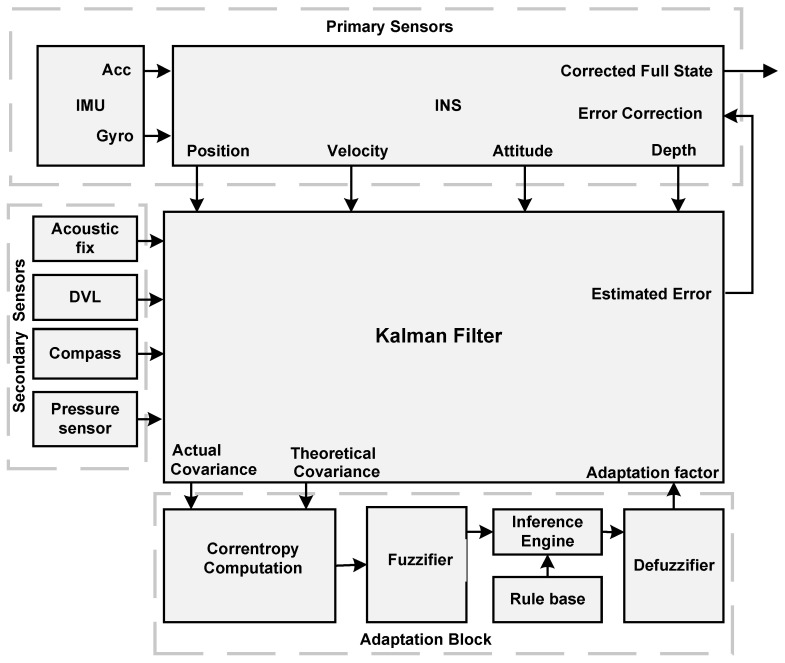
Top-level diagram of proposed integrated navigation architecture for underwater vehicle.

The proposed modifications improve the performance of Kalman filter-based multi-sensor fusion by utilizing the strengths of correntropy and fuzzy logic. Fuzzy logic has been shown to control nonlinear processes by using human linguistic expressions, and this capability is combined with Kalman filters to solve divergence problems and improve accuracy [4,32]. Covariance matching has been a popular methodology for adaptive fuzzy Kalman filtering in earlier research [8,33,50]; however, there is no reliable way for matching covariance when data have significant nonlinearity and heavy outliers, as in the case of an underwater vehicle navigation system. Therefore, we propose a correntropy-based covariance matching for fuzzy system input because of its robustness to outliers and non-Gaussian noise [51]. The top-level block diagram of our proposed multi-sensor fusion algorithm is depicted in Figure 2.

As shown by the above block diagram Figure 2, the primary sensors data from IMU and auxiliary sensors, such as the depth sensor, DVL, the acoustic position from USBL, and compass is fed into the Kalman-based fusion algorithm. The fusion algorithm works on the error dynamics of input data. The correntropy computation block is the part of the adaptation block that receives actual and theoretical covariance as input from the fusion block. The correntropy block provides a similarity measure from 0 to 1, where 1 means maximum similarity. The numerical value from the correntropy block is used for fuzzification. The fuzzified linguistic terms are passed through an inference engine that takes human expert-driven rules base. To obtain the adaption factor, the result of the inference engine is defuzzified. The correction is applied to covariance matrices which protect divergence and improve the accuracy of the filter.

### 4.1. Adaptation by Covariance Matching

The most commonly used approaches for adaptation are based on the covariance matching approach, which makes the theoretical value and actual value consistent with each other. The approach for adaptation of covariance matrices involves the simultaneous adaptation, both of the process and measurement noise covariances, or the adaptation of either of those two covariances if only one is known.

The theoretical covariance is used as a basis of comparison. It is given as

(20) $$S_{k}=H_{k}P_{k}^{-}H_{k}^{T}+R_{k}.$$

The actual covariance is calculated by taking a moving windows average of measurement innovation [15]. It is given as

(21) $$C_{k}=\frac{1}{\lambda }\sum_{i=i_{0}}^{\lambda }s_{k}s_{k}^{T},$$

where λ is the size of the window.

The most commonly used criteria used for fuzzy adaptive Kalman filter are DOM and DOD [35,36,52]. They are mathematically given by the following equations:

(22) $$DOD=Tr\left(S_{k}\right)-Tr\left(C_{k}\right)$$

(23) $$\mathrm{DOM}=S_{k}\left(j,j\right)-C_{k}\left(j,j\right).$$

The DOD gives one scalar value by subtracting the trace of theoretical and actual covariance matrices. On the other hand, DOM is a vector as a result of the diagonals difference of theoretical and actual covariance matrices.

When theoretical and actual covariance are perfectly matched, the DOD and DOM are close to zero. Moreover, the positive or negative values of these metrics indicate positive or negative direction of tuning for covariance matrices.

There are two major problems with this approach. Firstly, it ignores outliers and nonlinearity, both of which are frequent in underwater settings. Secondly, innovation’s autocorrelation does not reflect actual covariance. As a result, using DOM or DOD, the impact of outliers is reflected in the tuning process, which negatively influences the filter response. Furthermore, in the case of impulsive non-Gaussian noise, they do not provide accurate results.

### 4.2. Correntropy-Based Robust Adaptation of Process Noise Covariance by Gaussian Kernel

Correntropy is a similarity metric between two random variables [53]. It is based on kernel methods that take into account both a statistical distribution and temporal structure [9]. It is defined as

(24) $$M\left(A,B\right)=E\left\{\kappa \left(a_{k},b_{k}\right)\right\},$$

where κ is the kernel that satisfies Mercer conditions [54] and E is the expectation operator. Random variables are represented by A and B. In a practical situation, the joint distribution is not available and correntropy is calculated by using finite samples of random variables. It is calculated by using N samples of distributions as

(25) $$\hat{M}\left(A,B\right)=\frac{1}{N}\sum_{i=1}^{N}\kappa_{\sigma }\left(a_{k},b_{k}\right).$$

The most commonly used kernel is Gaussian, which reaches the maximum value when $a_{k}=b_{k}$. Moreover, the correntropy function based on the Gaussian is positive and bounded. It is written as

(26) $$\kappa_{\sigma }\left(a_{k},b_{k}\right)\;=\exp\left(-\frac{{∥a_{k}-b_{k}∥}^{2}}{2\sigma^{2}}\right),$$

where σ is the width of the kernel. The robustness will be good but the convergence speed will be slow if the kernel width is too small; conversely, if the kernel width is too large, the convergence speed will be rapid, but iterations may take a longer time. Selecting the appropriate kernel width is vital to the performance.

In this work, a new metric based on the correntropy Gaussian kernel is introduced to calculate a new comparison parameter named Degree of Convergence (DOC), which is the opposite of DOD and has better performance in nonlinear conditions. Furthermore, it solves two major problems by having the property to suppress the negative effects of the large outliers and providing better results in non-Gaussian conditions. The theoretical and actual covariance are matched using the DOC function, which is defined as

(27) $$DOC=\frac{1}{N}\sum_{i=1}^{N}\kappa_{\sigma }\left(e_{k}\right),$$

where $e_{k}$  is calculated as

(28) $$e_{k}=Tr\left(S_{k}\right)-Tr\left(C_{k}\right).$$

### 4.3. Fuzzification of Degree of Convergence

Fuzzification is the process of converting crisp values into fuzzy sets based on vague linguistic variables [55]. Fuzzification of DOC is based on its properties. In particular, DOC is maximum when theoretical and actual covariance is matched, and there is no need to tune Q. Furthermore, DOC is a symmetric positive function, therefore fuzzy variables are defined in a symmetric manner. Fuzzification of DOC used eight input linguistic terms: Positively Full Converge (PFC), Negatively Full Converge (NFC), Positively Moderate Converge (PMC), Negatively Moderate Converge (NMC), Positively Slight Converge (PSC), Negatively Slight Converge (NSC), Positively Diverge (PD), and Negatively Diverge (ND). The output linguistic variables are no change (NC), Moderate Decreased (MD), Moderate Increased (MI), Limited Decrease (LMD), Limited Increase (LMI), Significant Decrease (SD), and Significant Increase (SI). The fuzzy adaptation parameter for process noise covariance is donated by $\alpha_{k}$. The fuzzy rules for DOC are given as

- IF DOC is PFC THEN NC in $\alpha_{k}$
- IF DOC is NFC THEN NC in $\alpha_{k}$
- IF DOC is PMC THEN MD $\alpha_{k}$
- IF DOC is NMC THEN MI $\alpha_{k}$
- IF DOC is PSC THEN LMD $\alpha_{k}$
- IF DOC is NSC THEN LMI $\alpha_{k}$
- IF DOC is PD THEN SD in $\alpha_{k}$
- IF DOC is ND THEN SI in $\alpha_{k}$

A fuzzy set can be visually represented using membership functions. Different forms are determined by different sorts of mathematical formulas when expressing fuzzy sets with membership functions. The range [0, 1] is used to define fuzzy sets. The membership function of a fuzzy set J can be represented by

(29) $$J=\;\left\{\left(l,\mu_{J}\left(l\right)\right),\;\;\mathrm{such}\;\mathrm{that}\;l\in L\right\}.$$

The membership value of the element *l* in fuzzy subset J is denoted as $\mu_{J}\left(l\right)$. The universe *L* contains the crisp variable *l*.

The triangular function is defined by the following equations:

(30) $$\mu_{\dot{J}}\left(l\right)=\;\left\{\begin{array}{cc} 0, & l\leq a\\ \frac{l-a}{m-a}, & a<l\leq m\\ \frac{b-l}{b-m}, & \;m<l<b\\ 0, & l\geq b \end{array}\right.$$

where the lower limit is defined by *a*, an upper limit *b*, and *a* value *m*, where *a* < *m* < *b*.

The trapezoidal functions are used on the extreme left and right. The right trapezoidal function is defined as

(31) $$\mu_{J}\left(l\right)=\;\left\{\begin{array}{cc} 0, & l>d\\ \frac{d-l}{d-c}, & c\leq l\leq d\\ 1, & l<c \end{array}\right.$$

The left trapezoidal function is defined as

(32) $$\mu_{J}\left(l\right)=\;\left\{\begin{array}{cc} 0, & l<a\\ \frac{l-a}{b-a}, & a\leq l\leq b\\ 1, & l>b \end{array}\right.$$

Fuzzy inference is a method for determining how probable an input is to correspond to a specific output. The work utilizes Mamdani inferencing, which assumes that the output membership functions are fuzzy sets. Fuzzy “and” operation is computed Zadeh-min, taking the minimum of the two membership values.

Defuzzification is the process of transforming a fuzzy output, which cannot be used directly in a distinct crisp value. The center of gravity (COG) method [56] is used to get a crisp value of $\alpha_{k}$, given by the following equation

(33) $$\alpha_{k}=\frac{\sum_{i=1}^{n}\Delta_{i}\times e_{i}}{\sum_{i=1}^{n}\Delta_{i}},$$

where *n* depends on the partition of linguistic rules. $\Delta_{i}$ represent the area under the membership function (i), and $e_{i}$ is the *i*th centroid.

The adaptation of process noise covariance is given by the following equation [8,52]:

(34) $$Q_{k+1}=\left({g_{q}\alpha }_{k}+1\right){\tilde{Q}}_{k},$$

where ${\tilde{Q}}_{k}=diag\left[Q_{k}\right]$ and $g_{q}$ are gain scaling factors.

Algorithm 1 shows iterative steps for Q adaptation using fuzzy correntropy-based Kalman filtering by the Gaussian kernel.
**Algorithm 1 **Fuzzy Correntropy-based Kalman Filtering by Gaussian Kernel **  Initialize:**1:Initialization of KF state and covariance variables2:Initialization of fuzzy correntropy variables **  Time update:**3:Time update state
$x_{k+1}^{-}=\Phi_{k}x_{k}^{+}$4:Time propagation of covariance
$P_{k+1}^{-}=\Phi_{k}P_{k}^{+}\Phi_{k}^{⊤}+Q_{k}$ **  Calculation of Kalman gain:**5:Kalman gain:
$K_{k}=P_{k}^{-}H_{k}^{⊤}{\left(H_{k}P_{k}^{-}H_{k}^{⊤}+R_{k}\right)}^{-1}$ **  Innovation calculation:**6:The difference between measured and predicted value
$s_{k}=z_{k}-H_{k}x_{k}^{-}$ **  Measurement Update:**7:State is corrected by using Kalman gain and innovation
$x_{k}^{+}=\;x_{k}^{-}+K_{k}s_{k}$8:State covariance corrected by Kalman gain
$P_{k}^{+}=\;\left(I-K_{k}H_{k}\right)P_{k}^{-}$9:Theoretical covariance calculation
$S_{k}=H_{k}P_{k}^{-}H_{k}^{T}+R_{k}$10:Actual approximated covariance in moving windows
$C_{k}=\frac{1}{\lambda }\sum_{i=i_{0}}^{\lambda }s_{k}s_{k}^{T}$11:Correntropy based Degree of Convergence (DOC) calculation by using Gaussian kernel
$$DOC=\frac{1}{N}\sum_{i=1}^{N}\kappa_{\sigma }\left(s_{k},c_{k}\right)$$
$$\kappa_{\sigma }\left(s_{k},c_{k}\right)\;=\exp\left(-\frac{{∥e_{k}∥}^{2}}{2\sigma^{2}}\right)$$ **    Fuzzy adaptation of Q**12:Fuzzification using triangular and L and R type trapezoidal functions given in Equations (30)–(32).13:Application of rules using inference engine.14:Defuzzification to crisp output for $\alpha_{k}$ by Equation (33).15:Updated process noise covariance
$Q_{k+1}=\left({g_{q}\alpha }_{k}+1\right){\tilde{Q}}_{k}$16:Next iteration (posterior becomes prior)

### 4.4. Correntropy-Based Robust Adaptation of Measurement Noise Covariance Using Versoria Kernel

**Figure 3:**
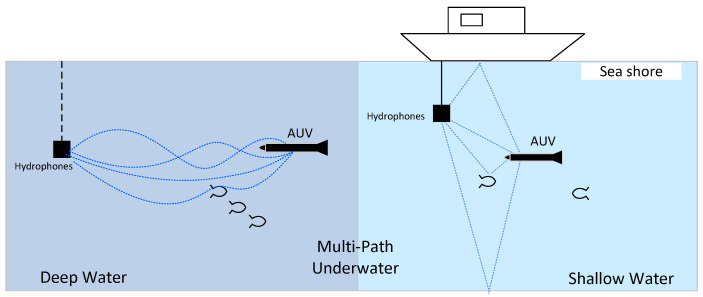
Multi-path effect in deep sea and shallow water.

Multi-path is one of the most challenging problems that acoustic systems face underwater. In shallow water, the signal propagates by reflections from the surface and bottom, in addition to the direct channel which causes the multi-path effect. On the other hand, in deep waters, a multi-path is created mainly as a result of ray bending due to refraction. Moreover, the sound speed profile which depends on temperature, depth, and salinity is also a critical contributor of multi-paths. Furthermore, air bubbles and marine animals have their parts for outliers. Figure 3 shows the multi-path in deep and shallow water.

The heavy-tailed distribution is a feasible representation for data that has been corrupted by multi-path outliers, which is common in underwater acoustics. The direct subtraction for covariance matching does not reduce the effects of outliers. These outliers’ heavier tail represents how the probability of extreme outcomes is higher in the tails than in the normal distribution. The Versoria correntropy kernel is well-suited in an underwater environment for its robustness to outliers. The tail of the Versoria function is heavier than Gaussian and Student’s t distributions [57]. Moreover, the Versoria kernel error converges faster than the exponential-based kernel. In addition, it has less computation complexity as compared to the Gaussian Kernel. We define another new metric called the Degree of Similarity (DOS), which is used to calculate correntropy using the Versoria function, given as:

(35) $$\mathrm{DOS}=\frac{1}{N}\sum_{i=1}^{N}\kappa_{\sigma }\left(\epsilon_{k}^{i}\right).$$

The Versoria function is given as

(36) $$\kappa_{\sigma }\left(\epsilon_{k}\right)=\frac{A^{3}}{A^{2}+{\left(\left|\epsilon_{k}^{i}\right|\right)}^{2}},$$

where A=2r and *r* are the radii of the circle located at (0,r) and $\epsilon_{k}$ represents the error, which is given as

(37) $$\epsilon_{k}^{i}=S_{k}^{i}\left(j,j\right)-C_{k}^{i}\left(j,j\right),$$

where $S_{k}\left(j,j\right)$, $C_{k}\left(j,j\right)$*i* to *N* samples are drawn from the storage of diagonal elements of the covariance matrix. The value of the DOS is positively bounded, and the direction of tuning is determined by the positive or negative sign of $\epsilon_{k}^{i}$.

The alternate representation of the Versoria function with the shaping factor τ is given as

(38) $$\kappa_{\sigma }\left(\epsilon_{k}\right)=\frac{2r}{1+\tau {\left(\left|\epsilon_{k}^{i}\right|\right)}^{2}},$$

where the shaping factor $\tau =\frac{1}{{\left(2r\right)}^{2}}$ is constant, and it is dependent on the diameter of the circle.

The adaptation of measurement noise is performed by the following equation [8,52]:

(39) $$R_{k+1}\left(j,j\right)=\zeta \left(j\right){\tilde{R}}_{k}\left(j,j\right),$$

where ${\tilde{R}}_{k}=diag\left[R_{k}\right]$ and ζ(j) are given as

(40) $$\zeta \left(j\right)=g_{r}\left(j\right)\gamma_{k}\left(j\right)+1,$$

where $g_{r}\left(j\right)$ is the scaling factor and $\gamma_{k}\left(j\right)$ is the fuzzy adaptation parameter for the *j*th element of the measurement noise covariance matrix.

### 4.5. Fuzzification of Degree Of Similarity

Six linguistic terms are defined as Positive Perfect Matched (PPM), Negative Perfect Matched (NPM), Positive Moderate Match (PMM), Negative Moderate Match (NMM), Positive Mismatch (PM), and Negative Mismatch (NMS). The positive and negative terms are defined by signs of the $\epsilon_{k}$. The output linguistics terms are defined as No Change (NC), Moderately Decrease (MOD), Moderately Increase (MOI), Large Decrease (LD), and Large Increase (LI).

### 4.6. Fuzzy Rules and Membership Functions

Fuzzy rules for DOS are defined by IF-THEN statements which are based on the knowledge of the system using linguistic variables. The rules draw conclusions based on one or more premises that act as an input to the system.

- IF DOS is PPM THEN NC in $\gamma_{k}$
- IF DOS is NPM THEN NC in $\gamma_{k}$
- IF DOS is PMM THEN MOD $\gamma_{k}$
- IF DOS is NMM THEN MOI $\gamma_{k}$
- IF DOS is PMS THEN LD in $\gamma_{k}$
- IF DOS is NMS THEN LI in $\gamma_{k}$

Fuzzy membership functions are defined using a combination of triangular and trapezoidal curves. The outer left and right are trapezoidal, and the inner curves are triangular. These curves have the advantage of faster processing time as compared to other types. The inference is used to assess how probable it is that an input correlates to a specific output; in this case, each of the rules employed only has one premise by utilizing a minimum fuzzy operator. Defuzzification is performed by the COG method, as discussed in the previous Section 4.3.

The Algorithm 2 shows iterative steps of measurement noise covariance R adaptation using fuzzy correntropy-based Kalman filtering by the Versoria kernel.
**Algorithm 2 **Fuzzy correntropy-based Kalman filtering by the Versoria kernel. **  Initialize:**1:Initialization of KF state and covariance variables2:Initialization of fuzzy correntropy variables **  Time update:**3:Time update state
$x_{k+1}^{-}=\Phi_{k}x_{k}^{+}$4:Time propagation of covariance
$$P_{k+1}^{-}=\Phi_{k}P_{k}^{+}\Phi_{k}^{⊤}+Q_{k}$$5:Kalman gain:
$K_{k}=P_{k}^{-}H_{k}^{⊤}{\left(H_{k}P_{k}^{-}H_{k}^{⊤}+R_{k}\right)}^{-1}$ **  Innovation calculation:**6:The difference between measured and predicted value
$s_{k}=z_{k}-H_{k}x_{k}^{-}$ **  Measurement update:**7:State is corrected by using Kalman gain and innovation
$x_{k}^{+}=x_{k}^{-}+K_{k}s_{k}$8:State covariance corrected by Kalman gain
$P_{k}^{+}=\;\left(I-K_{k}H_{k}\right)P_{k}^{-}$9:Theoretical covariance calculation
$S_{k}=H_{k}P_{k}^{-}H_{k}^{T}+R_{k}$10:Actual approximated covariance in moving windows
$C_{k}=\frac{1}{\lambda }\sum_{i=i_{0}}^{\lambda }s_{k}s_{k}^{T}$11:Correntropy based Degree of Similarity (DOS) calculation by using Versoria kernel
$$\mathrm{DOS}=\frac{1}{N}\sum_{i=1}^{N}\kappa_{\sigma }\left(\epsilon_{k}^{i}\right)$$
$$\kappa_{\sigma }\left(\epsilon_{k}\right)=\frac{2r}{1+\tau {\left(\left|\epsilon_{k}^{i}\right|\right)}^{2}}$$ **    Fuzzy adaptation of R**12:Fuzzification using triangular and L and R type trapezoidal functions given in Equations (30)–(32).13:Application of rules using inference engine.14:Defuzzification to crisp output for $\gamma_{k}$ is done by using Equation (33).15:Update measurement noise covariance
$R_{k+1}\left(j,j\right)=\zeta \left(j\right){\tilde{R}}_{k}\left(j,j\right)$16:Next iteration (posterior becomes prior)

**Figure 4:**
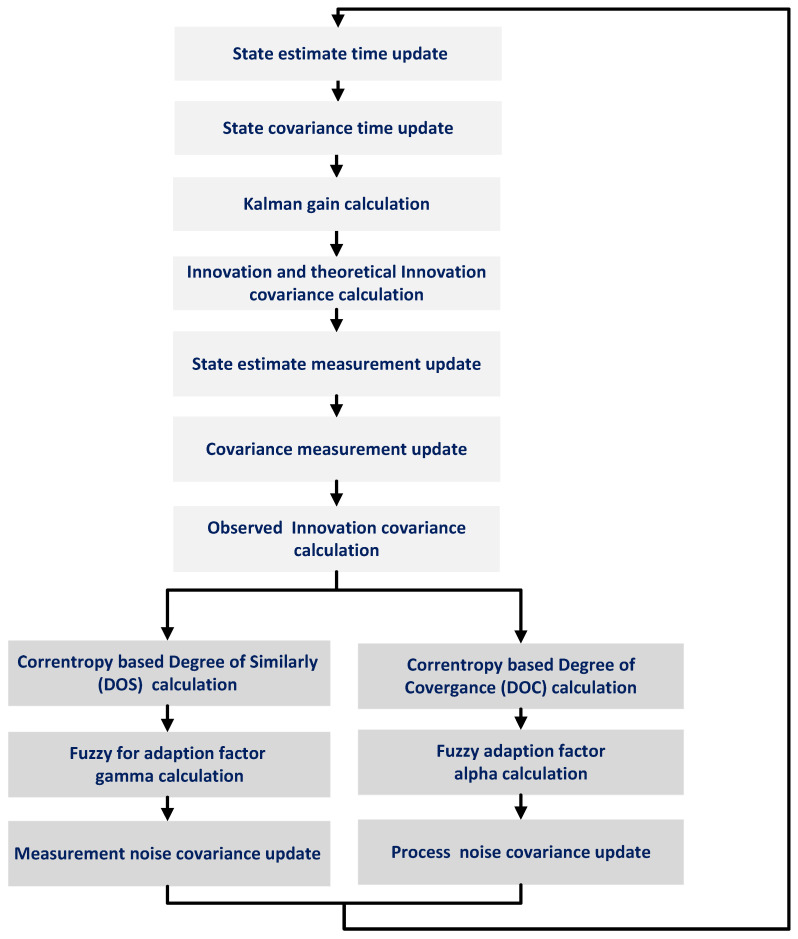
Flow chart representation of the proposed FC-MSF method.

Figure 4 shows a flow chart representation of Algorithm 1 and Algorithm 2 working together for process noise covariance and measurement noise covariance adaptation.

## 5. Simulation Results and Discussion

This section is divided into two subsections. First, Section 5.1 briefly explains the simulation scenario, and second, Section 5.2 discusses the simulation results.

### 5.1. Simulation Scenario

The performance of the proposed fuzzy correntropy-based multi-sensor algorithm is compared with Kalman-based multi-sensor fusion and fuzzy multi-sensor fusion using Monte Carlo simulation. The root mean square error (RMSE) was chosen as the main metric for comparison because it compares overall filter estimation performance over a longer length of time. Calculating the RMSE for the *i*th state of the estimated state vector $x_{est}$ and reference state $x_{ref}$ is given by

(41) $$RMSE=\sqrt{\frac{1}{n}\sum_{i=1}^{n}{\left(x_{ref}^{i}-x_{est}^{i}\right)}^{2}}$$

In order to assess the combined effects of the north, east, and down position and velocity, where the average *RMSE* is computed as

(42) $$\;\mathrm{Average}\;\mathrm{RMSE}=\frac{1}{3}\left({RMSE}^{north}+{RMSE}^{east}+{RMSE}^{down}\right).$$

The trajectory of the underwater vehicle is simulated by using different values of acceleration and angular velocities. All filters are tested on similar conditions with the same input data for valid comparison. The vehicle is assumed to be in the normal mode of operation, with no onboard or off-board sensor failures. The initial velocities of all three axes are zero. For position Initialization, the Latitude is initialized at $50^{\circ }$, Longitude is initialized at $5^{\circ }$, and height is initialized at zero. The attitude is represented in quaternion, and it is initialized at [1000] in the ECEF frame. The INS simulation model employs raw IMU measurement data and a starting position, velocity, and attitude to build underwater vehicle navigation profile. All the units used in this study are based on SI units. The position is represented in meters, and velocity in meters per second.

**Figure 5:**
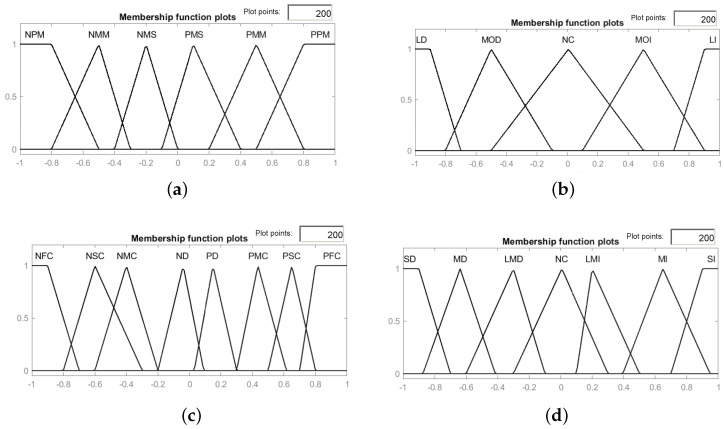
Fuzzy membership functions: (**a**) Input membership functions for DOS; (**b**) Output membership functions for DOS; (**c**) Input membership functions for DOC; (**d**) Output membership functions for DOC.

The input and output triangular and trapezoidal membership functions used in this work are displayed in Figure 5.

The design parameters for fuzzy logic are selected empirically, where the input gain for fuzzy scaling for process covariance adaptation is selected as 1, and output gain is selected as 0.001. For process noise adaptation, fuzzy input scaling is selected as 1 and the output is selected as 0.01. Another critical design parameter is the width of the kernel. In general, a wider kernel width provides a quicker convergence speed, but it generally results in worse shot noise performance. The kernel width of the Gaussian correntropy function is empirically selected as 0.01 and the Versoria shaping factor is chosen as 0.5.

The IMU was simulated at a data rate of 100 Hz, and the other secondary sensors at 10 Hz. For simulation of gyros bias and noise power spectral density are assumed to be $1^{\circ }/h$ and $0.1^{\circ }/s/\sqrt{\mathrm{Hz}}$ respectively. The accelerometers bias is considered to be 250 μg and noise power spectral density is 30 $\mu \sqrt{\mathrm{Hz}}$. The DVL standard deviation is assumed to be ±0.005m/s and random noise 0.1m/s, whereas the electronic compass was supposed to have bias $5^{\circ }$ and random noise $1^{\circ }$. Lastly, the depth sensor had 0.2m random noise.

The outliers were modeled as shot noise that is simulated by the amplitude of the noise which abruptly increases or decreases [39].

(43) $$w_{k}=\;\left(0,Q_{k}\right)\;+\;\mathrm{Shot}\;\mathrm{noise}\;$$

(44) $$v_{k}=\;\left(0,R_{k}\right)\;+\;\mathrm{Shot}\;\mathrm{noise}\;$$

### 5.2. Simulation Results

The performances of KF-MSF, Fuzzy (F-MSF) and correntropy-based fuzzy (FC-MSF) are compared in Table 1.

The RMSE of the position displayed in Table 1, it can be observed that estimation of KF-MSF has a very large error due to its inability to cover the correct position with the application of shot noise. However, F-MSF and FC-MSF have much lesser position errors as compared to KF-MSF. In comparison, the RMSE of the FC-MSF for the north and east positions is significantly better than F-MSF because it does not provide robust similarity measures as with correntropy-based similarity metrics. As compared to the north and east positions, the depth error is less since it does not suffer from random bias. Nevertheless, estimation of depth from FC-MSF is far better than KF-MSF and F-MSF.

The estimation of velocity from FC-MSF showed considerable improvement compared to the KF-MSF and FC-MSF. The overall northern velocity error was almost twice improved, like that of the F-MSF. Noticeable improvements were seen in east and downward velocities. The RMSE of the FC-MSF was found to be roughly twofold better than the ESKF for the north, east, and down velocities. Overall, with our proposed FC-MSF algorithm, the average of all RMSE velocities was almost two times better than F-MSF. In comparison to F-MSF and FC-MSF, the KF-MSF has inferior estimation performance due to the lack of a robust adaptation mechanism.

These results support the hypothesis that the correntropy-based fuzzy multi-sensor fusion is less susceptible to disturbances than the F-MSF and KF-MSF. Furthermore, the robustness property of the correntropy kernel allows the FC-MSF to perform better when shot noise is present. Moreover, FC-MSF estimation results are much better than position and velocity since the correntropy has the potential to capture high-order information. Conversely, conventional fuzzy Logic correction and KF-MSF without correntropy has a negative impact on the performance, given that these solutions are exposed to the same noise.

**Figure 6:**
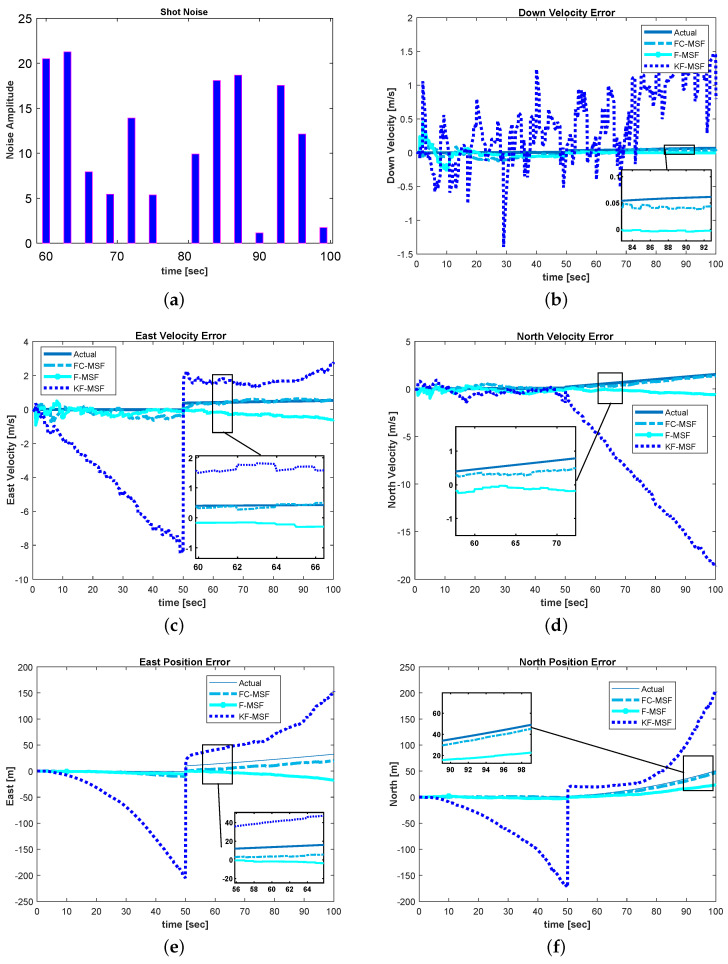
Simulation results with shot noise: (**a**) Shot noise amplitude added to the system; (**b**–**d**) show only FC-MSF velocities errors remain close to the actual, while KF-MSF significantly diverges from the actual value; (**e**,**f**) show position errors of the north and east, and it is evident that shot noise negatively influences KF-MSF and F-MSF position estimations.

Figure 6 shows a comparison of KF-MSF, F-MSF, and FC-MSF where we estimated the position and velocity.

The above-mentioned graphs clearly illustrate that FC-MSF has superior performance and errors are far less than KF-MSF and F-MSF. However, the suggested method is not restricted to underwater positioning applications; it may also be utilized to improve aerial positioning and navigation. Furthermore, autonomous cars can be another potential use for this approach. Additionally, satellite attitude estimates can be improved with the proposed technique.

## 6. Conclusions

This research study attempted to bridge a gap by providing a novel adaptive fusion method for underwater vehicle positioning by taking advantage of fuzzy and correntropy. The performance of the proposed algorithm is compared with Kalman and fuzzy-based sensor fusion techniques. It was found to have a better position and velocity estimation under the negative influence of shot noise. The primary aim of this work was to take advantage of correntropy and improve the covariance matching technique by using two new metrics, degree of similarity, and degree of convergence. The purpose was to improve the estimation performance of conventional methods for underwater vehicle positioning. The two proposed metrics help to enhance estimation accuracy through better matching of theoretical and actual covariance. The suggested technique is designed for use in underwater seabed mapping applications for ocean exploration.

## Figures and Tables

**Table 1:** Comparison of RMSE for position and velocity in the presence of shot noise by running 200 Monte-Carlo simulations with both R and Q adaptation.

RMSE	KF-MSF	F-MSF	FC-MSF
North Position	26.887	2.145	0.345
East Position	39.562	2.469	0.412
Down Position	9.513	0.353	0.051
Avg Position	25.321	1.655	0.269
North Velocity	1.608	0.388	0.146
East Velocity	1.529	0.485	0.121
Down Velocity	0.159	0.148	0.067
Avg Velocity	1.331	0.308	0.125

## Data Availability

Not applicable.
